# Clinical and high-resolution magnetic resonance imaging–based prediction of ischemic stroke in cervical artery dissection

**DOI:** 10.3389/fneur.2026.1860548

**Published:** 2026-06-24

**Authors:** Xuanxiao Zhang, Chunmei Liu, Shuo Yin, Xueliang Tian, Tao Li, Wenjing Lan, Hai Li, Hongwei Zhou

**Affiliations:** 1 Department of Radiology, The First Hospital of Jilin University, Changchun, China; 2 Changchun Obstetrics Gynecology Hospital, Changchun, China; 3 Department of Breast Surgery, General Surgery Center, The First Hospital of Jilin University, Changchun, China

**Keywords:** cervical artery dissection, high-resolution magnetic resonance imaging, intramural hematoma, ischemic stroke, nomogram

## Abstract

**Background:**

Cervical artery dissection (CeAD) is an important cause of ischemic stroke, yet early risk stratification remains challenging. This study aimed to identify clinical and high-resolution vessel wall magnetic resonance imaging features associated with ischemic stroke and to develop a patient-level model for short-term risk prediction.

**Methods:**

A total of 129 patients with CeAD (148 dissected vessels) were retrospectively included. Baseline clinical data and HRMRI features were collected. At the vessel level, least absolute shrinkage and selection operator (LASSO) regression was used for variable selection, followed by a mixed-effects logistic regression model to identify factors associated with ischemic events. At the patient level, representative vascular imaging features were integrated with clinical variables. LASSO regression and multivariable logistic regression were applied to construct a nomogram for risk prediction. Model performance was evaluated using receiver operating characteristic curves, calibration curves, and decision curve analysis.

**Results:**

At the vessel level, white blood cell (WBC) count, intraluminal thrombus, severe stenosis or occlusion, and alcohol consumption were independently associated with ischemic events. At the patient level, multivariable analysis showed that WBC count, intraluminal thrombus, male sex, and alcohol consumption were independent predictors of ischemic stroke. The nomogram exhibited good discriminative ability, with an optimism-corrected area under the curve of 0.837 (95% CI: 0.810–0.852), along with satisfactory calibration and clinical net benefit.

**Conclusion:**

A patient-level model shows good performance in predicting ischemic stroke risk in patients with CeAD and may assist in early risk stratification and individualized clinical decision-making.

## 1 Introduction

Cervical artery dissection (CeAD) is characterized by the formation of an intramural hematoma within the arterial wall, resulting from blood penetration into the intima-media layer following an intimal tear or vascular injury involving the carotid or vertebral arteries. This pathological process may subsequently lead to intraluminal thrombosis, vascular stenosis, occlusion, or the development of a dissecting aneurysm (1). CeAD represents a major cause of ischemic stroke in young and middle-aged adults, accounting for approximately 8–25% of ischemic strokes in individuals younger than 50 years (2, 3). Previous studies have reported that more than 50% of patients with CeAD develop ischemic stroke or transient ischemic attack (4, 5), with the highest stroke risk occurring within the first 2 weeks after diagnosis (6, 7). Therefore, early identification of patients at high risk for ischemic stroke is essential for optimizing clinical management and enabling individualized treatment strategies.

In recent years, high-resolution magnetic resonance imaging (HRMRI) has been increasingly recognized as a reliable and noninvasive imaging modality for the diagnosis and evaluation of CeAD (8, 9). HRMRI enables detailed visualization of the arterial wall and lumen, substantially improving the detection of intramural hematoma (IMH) (10–14). As a hallmark imaging feature of arterial dissection, IMH provides critical information for dissection classification and pathophysiological interpretation (15). Accumulating evidence suggests that IMH is closely associated with ischemic stroke in patients with CeAD, indicating that its imaging characteristics may play an important role in stroke risk stratification (14, 16). Nevertheless, current studies on the risk of ischemic stroke in patients with CeAD remain limited. Most previous studies have pooled heterogeneous imaging subtypes of CeAD and conducted analyses at either the single-vessel or individual-patient level, without adequately accounting for the statistical dependence introduced by multiple dissected vessels within the same patient. Consequently, patient-level risk prediction models that comprehensively reflect overall stroke risk remain scarce (17–19).

Therefore, based on HRMRI-derived imaging features and clinical data from patients with CeAD, this study aims to identify imaging and clinical characteristics associated with ischemic stroke risk at the patient level and to develop a nomogram for individualized risk prediction. In addition, vessel-level analysis is performed as an exploratory approach to investigate the association between imaging characteristics of individual dissected vessels and ischemic events, thereby providing supplementary insights into the mechanisms underlying dissection-related stroke.

## 2 Materials and methods

### 2.1 Patients

**Figure 1:**
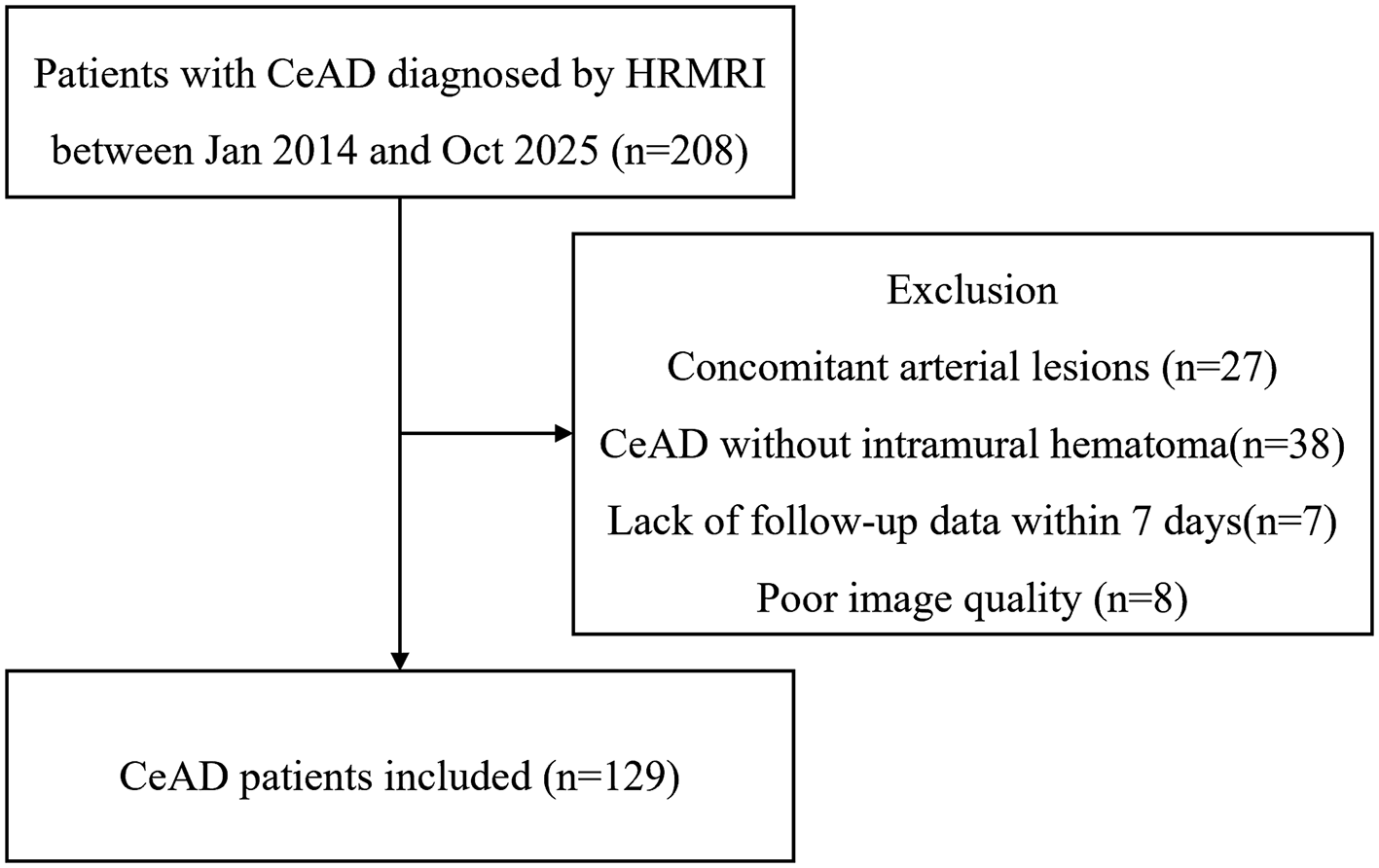
Flowchart of patient inclusion and exclusion.

This retrospective study was approved by the Ethics Committee of the First Hospital of Jilin University, and the requirement for informed consent was waived. Patients with CeAD who were admitted to our institution between January 2014 and October 2025 were retrospectively identified. Given the absence of a universally accepted gold standard for the diagnosis of CeAD, and considering that IMH demonstrates high imaging stability and diagnostic reliability on HRMRI, IMH was used as a key diagnostic criterion in this study to improve diagnostic consistency and reduce potential misclassification in retrospective imaging-based studies. In the present study, CeAD was diagnosed based on HRMRI findings demonstrating an IMH, with or without accompanying direct imaging signs, including a double-lumen sign, intimal flap, or dissecting aneurysm, in combination with clinical manifestations consistent with CeAD. The included patients were primarily symptomatic individuals hospitalized for evaluation of head or neck pain, acute neurological symptoms, or suspected cerebrovascular events. The inclusion criteria were as follows: (1) fulfillment of the above diagnostic criteria for CeAD; (2) availability of both head-and-neck HRMRI performed within 48 h of admission; (3) availability of clinical and imaging data for assessment of ischemic stroke within 7 days after admission. The exclusion criteria were as follows: (1) CeAD associated with other cerebrovascular diseases, including Moyamoya disease, vasculitis, fibromuscular dysplasia, or intracranial or carotid atherosclerosis with ≥50% stenosis or unstable plaques; (2) ischemic stroke attributable to cardioembolic sources; (3) poor image quality. The patient selection process is summarized in Figure 1. All clinical data were retrieved from the electronic medical record system, including demographic information, medical history, laboratory parameters, imaging characteristics, and treatment-related data.

### 2.2 Imaging protocol

All MRI examinations were performed on a 3.0-T MRI system (Philips Ingenia, Eindhoven, The Netherlands) using a 16-channel combined head-and-neck coil. HRMRI was acquired using a three-dimensional T1-weighted volume isotropic turbo spin-echo acquisition (3D T1 VISTA) sequence with the following parameters: repetition time = 350 ms; echo time = 19 ms; FOV = 280 × 199 × 120 mm^3^; matrix = 400 × 284; voxel size = 0.7 × 0.7 × 1.4 mm^3^; acquisition time = 3 min and 56 s.

### 2.3 Image analysis

All HRMRI images were independently evaluated by two radiologists with more than 5 and 10 years of diagnostic experience, respectively using the IntelliSpace Portal workstation, both of whom were blinded to the patients’ clinical information and DWI results. Image assessment was performed on original images and curved planar reformation (CPR) images. For each dissected vessel, the anatomical location (anterior or posterior circulation) and imaging features, including the double lumen sign, intimal flap, intraluminal thrombus, outward vessel dilatation, and atherosclerosis, were recorded. The length, degree of stenosis and signal characteristics of the IMH were measured. The double lumen sign was defined as blood flow separation into a true and a false lumen (20). An intimal flap was defined as a linear or curvilinear structure traversing the contrast-filled lumen and extending to the vessel wall on consecutive images (21, 22). Intraluminal thrombus was defined as high-signal intraluminal filling within the vascular lumen (23). IMH was identified as crescent-shaped thickening of the arterial wall. At the image slice showing the maximum thickness of the IMH, signal intensity was assessed using a semi-quantitative grading system as follows: extremely high signal, defined as signal intensity markedly higher than that of surrounding normal tissue; high signal, defined as signal intensity higher than surrounding normal tissue but lower than extremely high signal; and iso- or low signal, defined as signal intensity similar to or slightly lower than surrounding normal tissue (16). In addition, IMHs were classified as homogeneous or heterogeneous based on intralesional signal uniformity. The length of the IMH was measured on HRMRI CPR images. The degree of stenosis was assessed using NASCET method (24). The percentage of stenosis at the most severely narrowed segment was calculated as (1 − diameter of the diseased lumen/diameter of the reference lumen) × 100% and categorized as mild (<50%), moderate (50–69%), severe (≥70%), or complete occlusion.

### 2.4 Outcome definition

Primary analyses were performed at the patient level, while vessel-level data were retained for exploratory analyses to investigate the association between imaging features and ischemic events at different levels. At the patient level, the primary outcome was ischemic stroke identified within 7 days after admission, and patients were categorized according to the presence or absence of ischemic stroke. For vessel-level analyses, the study outcome was whether an individual dissected vessel was considered responsible for ischemic stroke. Based on DWI findings obtained during hospitalization, a dissected vessel was classified as responsible if acute infarct lesions were identified within its perfusion territory; otherwise, it was classified as non-responsible.

### 2.5 Statistical analysis

All statistical analyses were conducted using R software (v4.4.2; http://www.r-project.org). Continuous variables are presented as mean ± standard deviation or median (interquartile range), and categorical variables are expressed as frequencies (percentage). Categorical variables were analyzed using χ^2^ test or Fisher’s exact test, and continuous variables were compared using t-tests or Mann–Whitney *U* tests. All missing data were handled using multiple imputation, generating five complete datasets. Results from the imputed datasets were combined using Rubin’s rules. Inter-observer agreement for imaging features was evaluated using the intraclass correlation coefficient (ICC) for continuous variables and Fleiss’ kappa (*κ*) for categorical variables.

For patient-level analysis, when multiple dissected vessels were present, imaging characteristics and clinical variables from the vessel with the longest dissection length were selected as candidate features based on a predefined anatomical criterion, reflecting the most extensive vascular involvement. Univariate logistic regression was performed to describe the association between each candidate variable and ischemic stroke; these analyses were used for descriptive purposes only and did not guide variable selection. LASSO regression was subsequently performed separately within each imputed dataset to identify candidate predictors. A frequency-based selection approach was then applied to account for variability in variable selection across imputed datasets. Variables selected in more than 50% of the imputed datasets were retained for subsequent multivariable logistic regression analysis (25–27). Multicollinearity among variables in the final multivariable model was assessed using generalized variance inflation factors (GVIFs). A nomogram was then constructed to visually represent the combined effects of clinical and imaging features associated with ischemic stroke. To evaluate model performance, 1,000 bootstrap resamples were performed to estimate the optimism-corrected area under the curve (AUC). Calibration curves and decision curve analysis (DCA) were used to assess calibration and clinical utility. Additional sensitivity analyses were performed to evaluate the robustness of the primary findings, including analyses excluding patients with very short onset-to-admission intervals.

Vessel-level analysis was performed as an exploratory analysis. After LASSO regression-based variable selection, a mixed-effects logistic regression model was constructed, with individual vessels as analytical units and patient ID included as a random effect to account for within-patient correlations among vessels. Results of the vessel-level model are presented in the Supplementary material to illustrate potential associations between vascular imaging features and ischemic events. A two-sided *p*-value <0.05 was considered statistically significant.

## 3 Results

### 3.1 Patient characteristics

A total of 129 patients with CeAD were included, comprising 91 males and 38 females, aged 23–71 years (median, 41 years). According to follow-up results, 76 patients experienced ischemic stroke, while 53 patients did not. Baseline clinical and laboratory characteristics of all patients are summarized in Table 1.

**Table 1:** Baseline clinical and laboratory characteristics of patients with CeAD.

Characteristics	Overall (*n* = 129)	Nonstroke (*n* = 53)	Stroke (*n* = 76)	*p* value
Age, y (median [IQR])	41.00 [33.00, 53.00]	40.00 [34.00, 51.00]	42.50 [32.75, 55.00]	0.927
Male (%)	91 (70.54)	26 (49.06)	65 (85.53)	<0.001*
Head/neck pain (%)	54 (41.86)	26 (49.06)	28 (36.84)	0.167
Onset to admission time, d (median [IQR])	5.00 [2.00, 14.00]	7.00 [3.00, 14.00]	5.00 [2.00, 12.50]	0.171
Risk factors				
Hypertension (%)	47 (36.43)	13 (24.53)	34 (44.74)	0.190
Coronary heart disease (%)	14 (10.85)	6 (11.32)	8 (10.53)	0.887
Diabetes mellitus (%)	9 (6.98)	1 (1.89)	8 (10.53)	0.081
Hyperlipidemia (%)	49 (37.98)	18 (33.96)	31 (40.79)	0.432
Hyperhomocysteinemia (%)	23 (17.83)	5 (9.43)	18 (23.68)	0.037*
Hyperuricemia (%)	12 (9.30)	4 (7.55)	8 (10.53)	0.760
Smoking (%)	45 (34.88)	10 (18.87)	35 (46.05)	0.001*
Alcohol use (%)	38 (29.46)	6 (11.32)	32 (42.11)	<0.001*
Minor trauma history (%)	9 (6.98)	6 (11.32)	3 (3.95)	0.159
Stroke history (%)	10 (7.75)	4 (7.55)	6 (7.89)	1.000
Family history (%)	11 (8.53)	3 (5.66)	8 (10.53)	0.524
Recent infection (%)	16 (12.40)	5 (9.43)	11 (14.47)	0.393
Total number of vessels (median [IQR])	1.00 [1.00, 1.00]	1.00 [1.00, 1.00]	1.00 [1.00, 1.00]	0.780
Laboratory markers				
TC, mmol/L (median [IQR])	3.88 [3.34, 4.44]	3.88 [3.39, 4.36]	3.86 [3.32, 4.53]	0.711
TG, mmol/L (median [IQR])	1.23 [0.83, 1.78]	1.08 [0.76, 1.56]	1.28 [0.93, 1.91]	0.053
HDL-C, mmol/L(mean [SD])	1.14 (0.27)	1.18 (0.28)	1.12 (0.27)	0.220
LDL-C, mmol/L(median [IQR])	2.36 [1.96, 2.71]	2.39 [1.94, 2.71]	2.34 [1.98, 2.69]	0.670
Hcy, μmol/L (median [IQR])	11.70 [8.30, 15.40]	10.60 [8.10, 13.40]	12.61 [9.85, 17.92]	0.018*
WBC, ×10^9^/L (median [IQR])	7.55 [5.90, 8.97]	6.15 [5.19, 7.87]	8.28 [6.86, 9.75]	<0.001*
FIB, g/L (median [IQR])	2.87 [2.38, 3.38]	2.78 [2.31, 3.13]	2.91 [2.49, 3.45]	0.202

TC, total cholesterol; TG, triglyceride; HDL-C, high density lipoprotein-cholesterol; LDL-C, low density lipoprotein-cholesterol; Hcy, homocysteine; WBC, white blood cell; FIB, fibrinogen; IQR, interquartile range; SD, standard deviation. *p < 0.05.

### 3.2 Imaging characteristics of dissecting vessels

A total of 148 dissected vessels were included, of which 18 patients (13.9%) had multiple affected arteries. Among the 148 vessels, 42 (28.4%) were located in the internal carotid artery, and 106 (71.6%) were located in the vertebral artery. Of these, 82 vessels (55.4%) were classified as responsible for ischemic stroke, while 66 vessels (44.6%) were non-responsible.

### 3.3 Selection of patient-level variables

**Figure 2:**
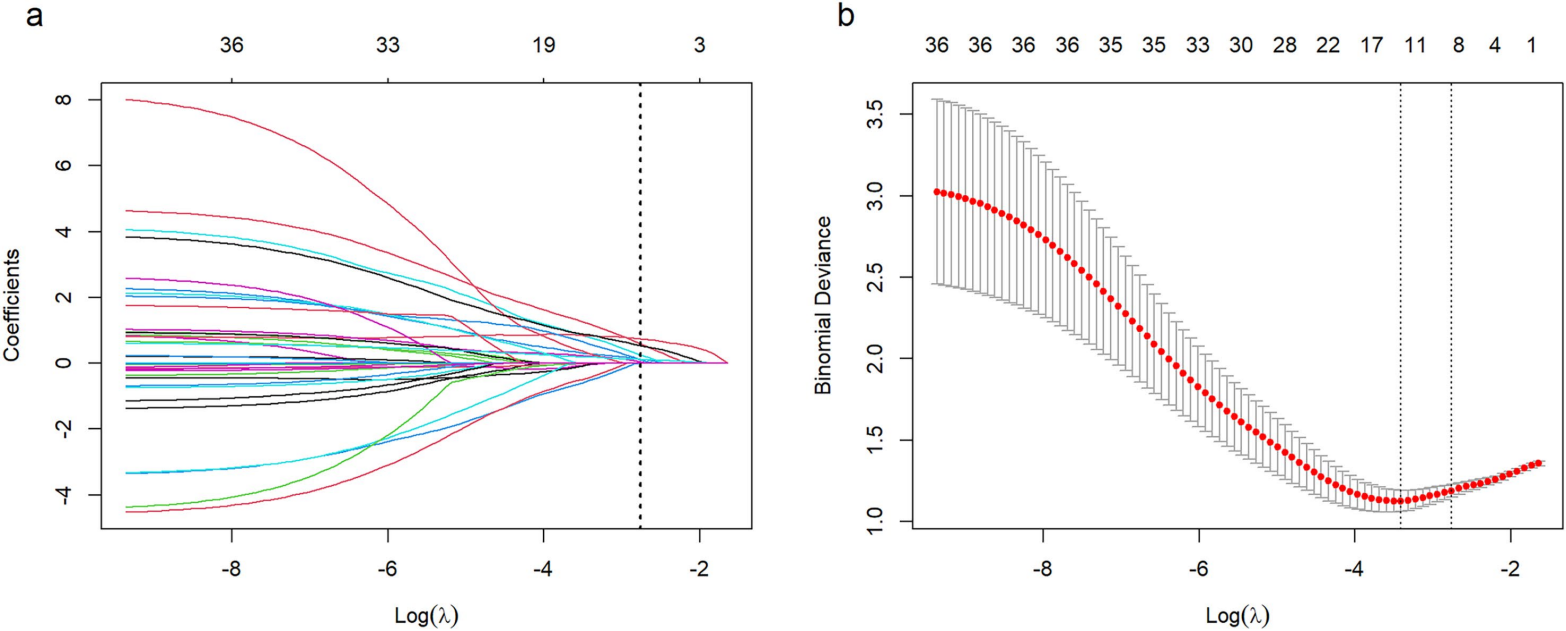
Variable Selection Based on the LASSO Regression Model. **(A)** Coefficient profiles of the LASSO logistic regression model. **(B)** Selection of the penalty parameter (*λ*) based on the 10-fold cross-validation curve. The left vertical line indicates the minimum criterion, whereas the right vertical line represents the 1-SE criterion used for variable selection.

Univariate logistic regression analyses were performed on multiple imputed datasets, and results were combined using Rubin’s rules, with detailed results shown in the Supplementary Table 1. Candidate patient-level variables were subsequently screened using LASSO regression, ultimately identifying six variables as potential predictors of ischemic stroke: intraluminal thrombus, stenosis degree, hematoma signal, male sex, alcohol consumption, and white blood cell count (Figure 2). No significant multicollinearity was observed among variables in the final multivariable model, with adjusted GVIF values ranging from 1.02 to 1.11, indicating a low degree of collinearity. Detailed results are provided in the Supplementary Table 3.

### 3.4 Development of the nomogram model

**Figure 3:**
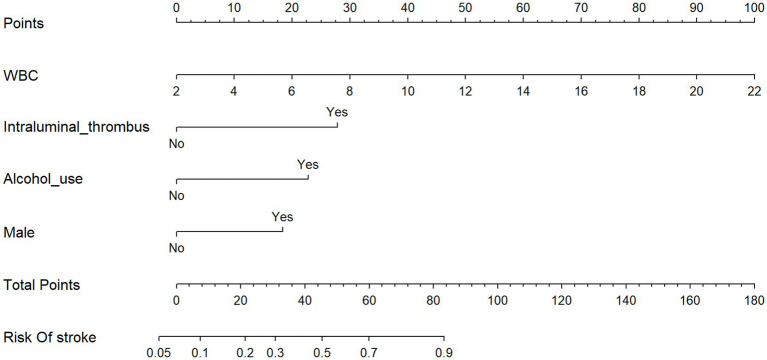
Nomogram for predicting the risk of ischemic stroke based on a multivariable logistic regression model.

**Figure 4:**
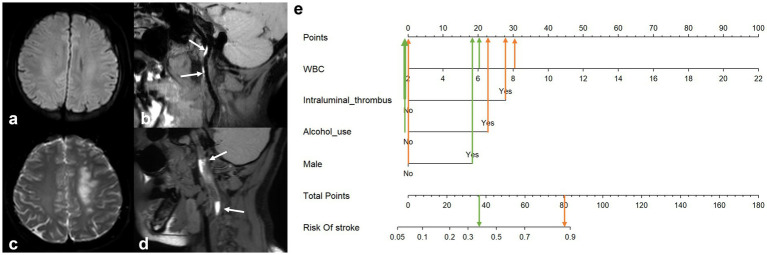
Clinical examples illustrating the application of the nomogram. **(A, B)** Male patient, 39 years old, no history of alcohol consumption, WBC 6.03 × 10^9^/L. **(A)** DWI shows no ischemic stroke. **(B)** HRMRI CPR image shows dissection of the left internal carotid artery; the arrow indicates the intramural hematoma. **(C, D)** Female patient, 49 years old, with a history of alcohol consumption, WBC 8.06 × 10^9^/L. **(C)** DWI demonstrates ischemic stroke in the left centrum semiovale. **(D)** HRMRI CPR image shows dissection of the left internal carotid artery; arrows indicate the intramural hematoma and intraluminal thrombus. **(E)** Nomogram scores: green arrow indicates the non-stroke patient, and orange arrow indicates the stroke patient.

Multivariable logistic regression analysis identified white blood cell (WBC) count (OR, 1.29; 95% CI, 1.06–1.57; *p* = 0.014), intraluminal thrombus (OR, 7.51; 95% CI, 1.87–30.12; *p* = 0.005), male sex (OR, 3.69; 95% CI, 1.27–10.72; *p* = 0.018), and alcohol consumption (OR, 3.52; 95% CI, 1.09–11.36; *p* = 0.037) as independent predictors of acute ischemic stroke (Table 2). In a sensitivity analysis excluding patients with onset-to-admission time <24 h, WBC count remained positively associated with acute ischemic stroke, although the statistical significance approached the borderline level (*p* = 0.054). Intraluminal thrombus and male sex remained statistically significant, while alcohol consumption showed a similar directional association. Overall, the findings of the sensitivity analysis were largely consistent with those of the primary analysis (Supplementary Table 4). A nomogram based on the multivariable logistic regression model (Figure 3) was constructed to facilitate individualized prediction of ischemic stroke risk. Clinical examples illustrating the application of the nomogram are presented in Figure 4.

**Table 2:** Results of multivariable logistic regression analysis at the patient level (*n* = 129).

Variable	OR (95% CI)	*p* value
Male	3.69 (1.27, 10.72)	0.018*
Alcohol use	3.52 (1.09, 11.36)	0.037*
WBC	1.29 (1.06, 1.57)	0.014*
Intraluminal thrombus	7.51 (1.87, 30.12)	0.005*
Stenosis degree		
Moderate	1.99 (0.55, 7.19)	0.298
Severe/occlusion	2.59 (0.82, 8.23)	0.108
Hematoma signal		
Hyperintense	0.68 (0.13, 3.46)	0.643
Very hyperintense	2.50 (0.65, 9.67)	0.186

WBC, white blood cell. *p < 0.05.

### 3.5 Performance of the nomogram model

**Figure 5:**
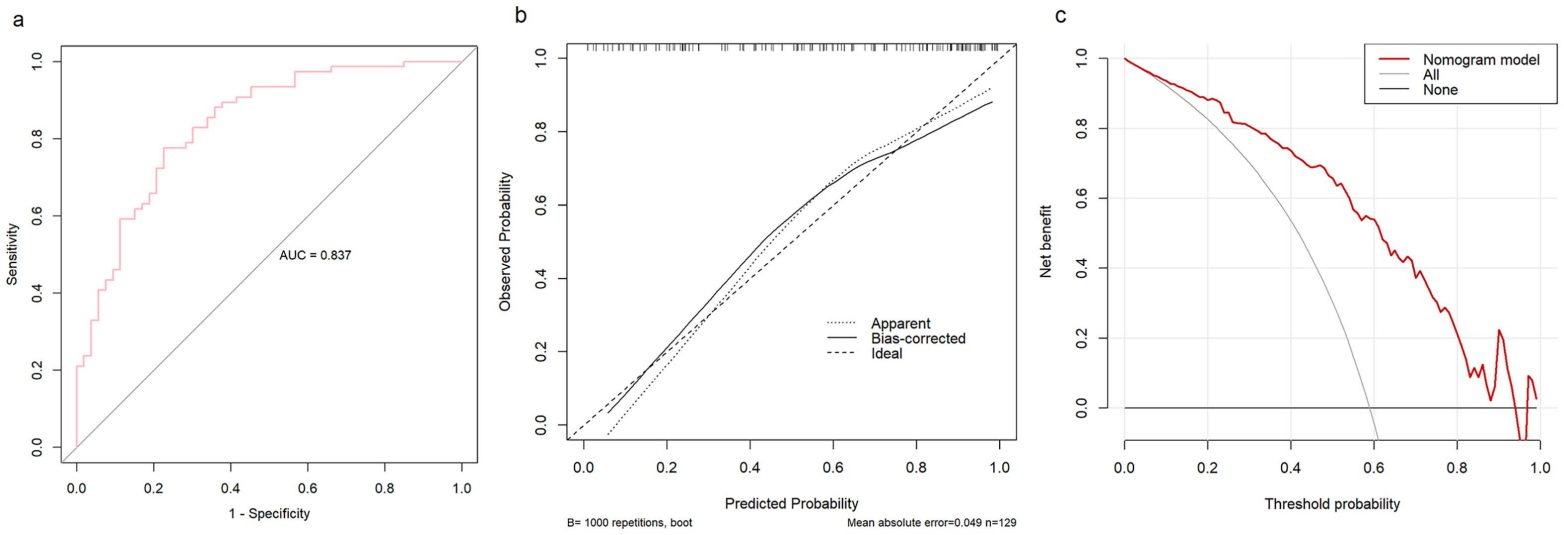
Performance of the nomogram model. **(A)**: ROC curve of the nomogram model (AUC = 0.837); **(B)**: Calibration curve of the nomogram model (mean absolute error = 0.049); **(C)**: Clinical decision curve of the nomogram model.

The discriminative ability of the patient-level logistic regression model was assessed using the ROC curve, yielding an apparent AUC of 0.855 (95% CI, 0.786–0.919). Internal validation with 1,000 bootstrap resamples resulted in an optimism-corrected AUC of 0.837 (95% CI, 0.810–0.852), indicating robust discrimination. The calibration curve demonstrated good agreement between predicted and observed outcomes, with a mean absolute error of 0.049. The optimism-corrected calibration intercept and slope were 0.054 and 0.763, respectively. The decision curve analysis showed that the model provided a net clinical benefit across a threshold probability range of 5 to 95% (Figure 5).

### 3.6 Agreement of measurements

Interobserver reproducibility was excellent for identifying intimal flaps, double lumen sign, intraluminal thrombus, vessel dilatation, atherosclerosis, and IMH homogeneity, with Kappa values of 0.900, 0.837, 0.920, 0.926, 0.916, and 0.825, respectively. The ICC for IMH length was 0.934, with all *p* values < 0.05. Detailed agreement statistics, including 95% confidence intervals and *p*-values, are provided in the Supplementary Table 5.

## 4 Discussion

This study systematically investigated factors associated with ischemic stroke secondary to CeAD at both the patient and vessel levels using high-resolution MRI and clinical data from patients with CeAD. At the patient level, intraluminal thrombus, male sex, alcohol consumption, and white blood cell count were identified as factors independently associated with ischemic stroke. A nomogram integrating these variables showed good discriminatory performance and calibration, serving as a visual summary of imaging and clinical factors associated with ischemic risk. Exploratory vessel-level analysis further demonstrated significant associations of white blood cell count, intraluminal thrombus, severe vessel stenosis or occlusion, and alcohol consumption with ischemic events in individual dissected vessels.

IMH, one of the characteristic imaging features of CeAD, can be clearly visualized on HRMRI. The progression of IMH may lead to intraluminal thrombosis, luminal stenosis, or the formation of a dissecting aneurysm, thereby increasing the risk of ischemic events (1, 12). McNally et al. reported an association between IMH and ischemic stroke in patients with CeAD, highlighting the potential clinical relevance of IMH-related imaging characteristics for stroke risk assessment (14). Moreover, the IMH-predominant subtype of CeAD has been shown to be particularly informative for imaging-based evaluation, risk stratification, and individualized management strategies (15). Accordingly, IMH-based HRMRI assessment may provide additional value for ischemic stroke risk assessment in patients with CeAD.

In exploratory vessel-level analyses, certain imaging features exhibited differences in statistical significance compared with patient-level analyses. For example, vessel stenosis was significantly associated with ischemic events at the vessel level but did not remain an independent factor in the patient-level model. This discrepancy likely reflects the distinct biological and clinical perspectives captured by the two analytical levels. Severe stenosis or occlusion of an individual vessel may cause distal hypoperfusion or facilitate local thrombus formation, thereby increasing ischemic risk at the vessel level (28, 29). However, at the patient level, the impact of these localized lesions may be modulated by multiple factors, thereby attenuating their independent contribution to overall stroke risk. Previous studies have demonstrated that severe vascular stenosis and poor collateral circulation are associated with an increased risk of ischemic events in patients with spontaneous internal carotid artery dissection, whereas favorable collateral circulation may confer a protective effect (30). Nevertheless, the underlying mechanisms warrant further investigation. In contrast, sex represents a systemic biological attribute rather than a localized vascular characteristic, which may explain its stronger association with stroke risk at the patient level. These findings suggest that different variables may play different roles in local vessel-related ischemic events and overall stroke status at the patient level.

Consistent with prior studies, our results support thromboembolism as a predominant mechanism of ischemic stroke in patients with CeAD (31). Liu et al. (32) demonstrated that HRMRI enables comprehensive visualization of vessel wall morphology and thrombus burden, providing strong evidence for the association between non-occlusive intraluminal thrombus and ischemic stroke occurrence. Multiple HRMRI-based studies have similarly reported intraluminal thrombus as a key imaging marker associated with ischemic events in CeAD (17, 33, 34). In addition, accumulating evidence highlights the important role of inflammation in the pathogenesis of both CeAD and ischemic stroke (35–41). WBC count, as a readily available marker of systemic inflammatory status, may reflect leukocyte-mediated vascular injury, thrombus formation, and subsequent ischemic events (41). In this study, WBC count showed a consistent association across different analytical levels, which is in line with the findings of Zhao et al. (36), who reported that elevated WBC count was associated with ischemic stroke occurrence in patients with carotid artery dissection. However, as elevated WBC levels are also commonly observed as part of the secondary inflammatory response following acute ischemic stroke, the possibility of reverse causality cannot be completely excluded. To further address this concern, we performed an additional sensitivity analysis excluding patients with onset-to-admission time <24 h. The association between WBC count and acute ischemic stroke became borderline significant after exclusion, although the direction of the association remained consistent with the primary analysis. This finding suggests that the observed association may be partially influenced by inflammatory responses following very early ischemic injury, while WBC may still retain predictive value as a risk-associated marker. Therefore, WBC should be interpreted as a risk-associated marker rather than a direct causal factor for ischemic stroke in CeAD. Prospective studies are warranted to further clarify the temporal relationship between inflammatory response and stroke occurrence.

In clinical practice, antiplatelet agents and anticoagulation are commonly used as antithrombotic strategies in patients with CeAD to reduce the risk of thromboembolic events; however, the optimal treatment strategy remains controversial. In this study, all included patients received individualized standard antithrombotic therapy after admission based on clinical assessment, imaging findings, and current guideline recommendations. Antithrombotic therapy was considered as a candidate variable during the variable selection process but was not retained in the LASSO selection and was therefore not included in the final predictive model.

The nomogram developed in the present study may have potential value for individualized early ischemic stroke risk assessment in patients with CeAD. By integrating HRMRI imaging features with clinical variables, the model may facilitate early identification of patients at higher risk of ischemic events and enable more refined risk stratification. Such stratification may provide supportive information for clinical monitoring and follow-up strategies. In addition, incorporation of HRMRI findings into the model may contribute to more comprehensive risk assessment and further support the potential role of imaging biomarkers in CeAD.

This study has several limitations. First, this was a single-center retrospective study with a relatively small sample size and without an independent external validation cohort. Although bootstrap internal validation was performed to reduce potential optimism bias, further validation is still required to evaluate the generalizability and robustness of the model. Future prospective multicenter studies with larger sample sizes and independent external validation cohorts are warranted to further evaluate the predictive performance and clinical utility of the proposed nomogram. Second, the patient-level model incorporated imaging characteristics from only a single representative vessel, which may not fully capture the cumulative risk associated with multiple dissected vessels and could lead to underestimation of overall ischemic risk in some patients. Third, several imaging-related limitations should be acknowledged. The study population was restricted to patients with visible IMH on HRMRI, which may introduce selection bias and limit generalizability. As a dynamic imaging marker, IMH may exhibit temporal variations in extent and signal intensity; thus, reliance on a single time-point assessment may affect the accuracy of stroke risk prediction.

## 5 Conclusion

In conclusion, this study identified several clinical and HRMRI features associated with ischemic stroke in CeAD from both patient- and vessel-level perspectives. The proposed nomogram demonstrated good discriminative ability and calibration, and may serve as a potential tool for individualized risk assessment. These findings provide additional insights into risk stratification and may contribute to a better understanding of the underlying mechanisms of ischemic stroke in CeAD.

## Data Availability

The raw data supporting the conclusions of this article will be made available by the authors, without undue reservation.
