# Supplementary material for: Clinical and high-resolution magnetic resonance imaging–based prediction of ischemic stroke in cervical artery dissection
Source: Front Neurol. 2026 Jun 24;17:1860548. doi: 10.3389/fneur.2026.1860548 (PMC13341283; doi:10.3389/fneur.2026.1860548)
Supplement: Supplementary file 1 [file Data_Sheet_1.docx]

Supplementary Material

**Supplementary Vessel-Level Analysis**

The intraclass correlation coefficient (ICC) ranged from 0.19 to 0.26 across imputed datasets, indicating the presence of within-patient clustering effects among vessels (Supplemental Table 6). LASSO regression identified six candidate variables associated with ischemic vascular events, including white blood cell (WBC) count, hypertension, intraluminal thrombus, stenosis degree, male sex, and alcohol consumption (Supplemental Figure 1). These variables were subsequently entered into a mixed-effects logistic regression model. Multivariable analysis revealed that elevated WBC count, intraluminal thrombus, and severe vessel stenosis or occlusion, as well as alcohol consumption, were independently associated with ischemic vascular events (Supplemental Table 2).

# Supplementary Figure


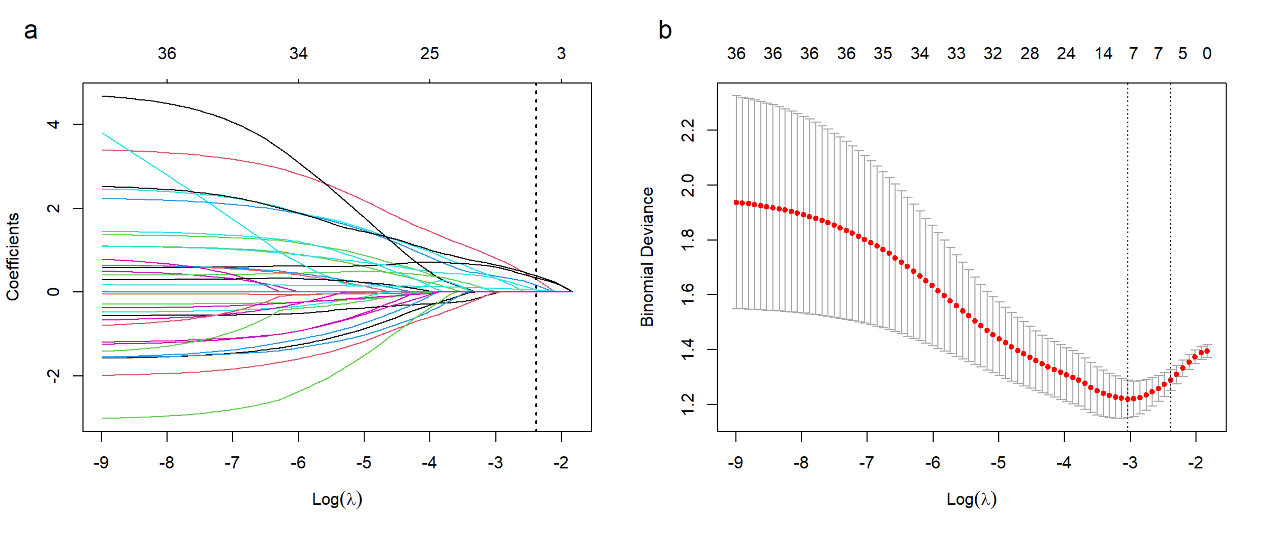


**Supplemental Figure 1**: Variable Selection for the Vessel-Level Analysis Using LASSO Regression.

(a) Coefficient profiles of the LASSO logistic regression model. (b) Selection of the penalty parameter (λ) based on the 10-fold cross-validation curve. The left vertical line indicates the minimum criterion, whereas the right vertical line represents the 1-SE criterion used for variable selection.

# Supplementary Tables

**Supplementary Table 1.**  Results of Univariate Logistic Regression Analysis at the Patient Level（n=129）

| **Variable** | **OR (95% CI)** | **P value** |
| --- | --- | --- |
| Age | 1.01 (0.98, 1.04) | 0.638 |
| Male | 6.14 (2.64, 14.27) | ＜0.001* |
| Head/neck pain | 0.61 (0.30, 1.24) | 0.170 |
| Onset to admission time | 1.00 (0.98, 1.01) | 0.888 |
| **Risk factors** |  |  |
| Hypertension | 2.49 (1.14, 5.43) | 0.022* |
| Coronary heart disease | 0.92 (0.30, 2.86) | 0.887 |
| Diabetes mellitus | 6.12 (0.73, 51.51) | 0.095 |
| Hyperlipidemia | 1.34 (0.64, 2.80) | 0.434 |
| Hyperhomocysteinemia | 2.98 (1.02, 8.71) | 0.046* |
| Hyperuricemia | 1.44 (0.41, 5.12) | 0.569 |
| Smoking | 3.67 (1.60, 8.42) | 0.002* |
| Alcohol use | 5.70 (2.15, 15.08) | 0.001* |
| Minor trauma history | 0.32 (0.08, 1.37) | 0.124 |
| Stroke history | 1.05 (0.28, 3.97) | 0.942 |
| Family history | 1.96 (0.49, 7.87) | 0.339 |
| Recent infection | 1.62 (0.52, 5.04) | 0.398 |
| Total number of vessels | 0.96 (0.37, 2.45) | 0.927 |
| **HRMRI features** |  |  |
| Anterior circulation | 1.07 (0.50, 2.30) | 0.867 |
| Length | 0.98(0.84, 1.15) | 0.847 |
| Double lumen | 0.92 (0.45, 1.89) | 0.817 |
| Intimal flap | 0.69 (0.29, 1.64) | 0.404 |
| Hematoma homogeneity | 0.53 (0.20, 1.39) | 0.195 |
| Intraluminal thrombus | 4.68 (1.48, 14.74) | 0.009* |
| Vessel dilatation | 1.10 (0.54, 2.24) | 0.785 |
| Atherosclerosis | 1.54 (0.65, 3.65) | 0.328 |
| Hematoma signal |  |  |
| Very hyperintense | 1.98 (0.72, 5.43) | 0.183 |
| Hyperintense | 1.01 (0.29, 3.54) | 0.987 |
| Stenosis degree |  |  |
| Moderate | 2.22 (0.79, 6.22) | 0.128 |
| Severe/Occlusion | 4.06 (1.62, 10.16) | 0.003* |
| **Laboratory markers** |  |  |
| WBC | 1.35 (1.12, 1.62) | 0.001* |
| TC | 0.89 (0.62, 1.26) | 0.494 |
| TG | 1.40 (0.82, 2.40) | 0.212 |
| HDL-C | 0.37 (0.10, 1.43) | 0.148 |
| LDL-C | 0.90 (0.58, 1.41) | 0.641 |
| Hcy | 1.03 (0.98, 1.09) | 0.203 |
| FIB | 1.04 (0.72, 1.49) | 0.837 |

Note: TC, total cholesterol; TG, triglyceride; HDL-C, high density lipoprotein-cholesterol; LDL-C, low density lipoprotein-cholesterol; Hcy, homocysteine; WBC, white blood cell; FIB, fibrinogen; IQR, interquartile range; SD, standard deviation. *P < 0.05.

**Supplementary Table 2.** Results of mixed-effects logistic regression at the vessel level

| **Variable** | **OR (95% CI)** | **P value** |
| --- | --- | --- |
| Male | 2.63 (0.72, 9.58) | 0.142 |
| Alcohol Use | 4.21 (1.04, 17.09) | 0.045* |
| WBC | 2.01 (1.02, 3.97) | 0.044* |
| Hypertension | 1.89 (0.65, 5.50) | 0.239 |
| Intraluminal Thrombosis | 6.82 (1.27, 36.63) | 0.026* |
| Stenosis Degree |  |  |
| Moderate | 3.56 (0.87, 14.53) | 0.077 |
| Severe/Occlusion | 5.79 (1.39, 24.04) | 0.016* |

Note: WBC, white blood cell. *P < 0.05.

**Supplementary Table 3.** Adjusted GVIF Values for Variables in the Final Patient-Level Model

| **Variable** | **Adjusted GVIF** |
| --- | --- |
| WBC count | 1.04 |
| Intraluminal thrombus | 1.05 |
| Stenosis degree | 1.02 |
| Male sex | 1.11 |
| IMH signal | 1.06 |
| Alcohol consumption | 1.10 |

**Supplementary Table 4.** Multivariable logistic regression analysis in the sensitivity cohort excluding patients with onset-to-admission time <24 hours

| **Variable** | **OR (95% CI)** | **P value** |
| --- | --- | --- |
| Male | 5.46 (1.71–17.49) | 0.005* |
| Alcohol Use | 2.67 (0.78–9.18) | 0.059 |
| WBC | 1.24 (1.00–1.54) | 0.054 |
| Intraluminal Thrombus | 4.95 (1.20–20.47) | 0.030* |
| Stenosis Degree |  |  |
| Moderate | 1.81 (0.47–7.04) | 0.393 |
| Severe/Occlusion | 3.28 (0.97–11.13) | 0.123 |
| Hematoma Signal |  |  |
| Hyperintense | 1.26 (0.21–7.55) | 0.798 |
| Very Hyperintense | 3.30 (0.79–13.70) | 0.103 |

Note: WBC, white blood cell. *P < 0.05.

**Supplementary Table 5.** Inter-observer agreement analysis of imaging features

| **Variables** | **ICC or Kappa** | **95% CI** | ***p* value** |
| --- | --- | --- | --- |
| Intimal flaps | 0.900 | 0.711-0.989 | <0.001 |
| Double lumen sign | 0.837 | 0.759-0.915 | <0.001 |
| Intraluminal thrombus | 0.920 | 0.767-1 | <0.001 |
| Vessel dilatation | 0.926 | 0.784-1 | <0.001 |
| Atherosclerosis | 0.916 | 0.753-1 | <0.001 |
| IMH homogeneity | 0.825 | 0.706-0.939 | <0.001 |
| IMH length | 0.934* | 0.857-0.930 | <0.001 |

* ICC.

**Supplementary Table 6.** Intraclass Correlation Coefficients Across Multiple Imputed Datasets

| **Imputed dataset** | **ICC** |
| --- | --- |
| 1 | 0.226 |
| 2 | 0.229 |
| 3 | 0.211 |
| 4 | 0.189 |
| 5 | 0.260 |
